# Boiled Milk

**Published:** 1897-06

**Authors:** 


### BOILED MILK.

Practical and every day experience shows that when milk is boiled it is not only more easily digested, but that it has a nutritive value quite equal to the raw article. Experiments undertaken by Dr. C. Chamouin (*Canadian Lancet*), first with kittens and afterwards with infants, showed after exhaustive and repeated trials that the kittens fed on boiled milk were "twice again as fat" as those supplied with the raw milk, and that the boiling of milk is the means of preventing the loss of innumerable lives by gastro-intestinal disease. Not only so, but it is more easily digested, and agrees with a far greater percentage of cases than unboiled milk. There is ample authority for this view of the case, but certain points must be attended to, else the results will not be so favorable. First, all the vessels in which the milk is carried, boiled, and afterwards kept must be scrupulously clean. Nothing else but absolute freedom from dirt will suffice. Then is should never be boiled in an open vessel; this should have a close cover. Lastly, it need not be kept at 212° F. for more than twenty minutes. This is sufficient to sterilize and cook it and no further boiling is necessary.—*Practitioner.*

